# Prevalence and factors associated with self-reported anxiety in adults during the COVID-19 pandemic in Argentina, Brazil, Peru, Mexico, and Spain: A cross-sectional Ibero-American study

**DOI:** 10.1371/journal.pone.0280528

**Published:** 2023-03-02

**Authors:** Gabriela Oliveira, Fernanda Garcia Gabira Miguez, Oscar G. Enríquez-Martinez, Taisa S. S. Pereira, Karen Villaseñor Lopez, Salomon Huancahuire-Vega, Marcia C. T. Martins, Sandaly O. S. Pacheco, Fabio J. Pacheco, Maria P. M. López, Maria del Carmen Bisi Molina

**Affiliations:** 1 Public Health Program, Health Sciences Center, Federal University of Espírito Santo, Vitória, Espírito Santo, Brazil; 2 Postgraduate Program in Nutrition and Health, Health Science Center, Federal University of Espírito Santo, Vitória, Brazil; 3 Department of Health Sciences, Universidad de las Américas Puebla, San Andrés Cholula, Puebla, Mexico; 4 Department of Basic Sciences, Faculty of Health Sciences, School of Human Medicine, Universidad Peruana Unión, Lima, Peru; 5 Interdisciplinary Center for Research in Health and Behavioral Sciences, School of Medicine, Universidad Adventista del Plata, Entre Ríos, Argentina; 6 Master in Human Motricity Sciences, Universidad Adventista de Chile, Chillán, Chile; 7 Biology Department, Universidad Autónoma de Madrid, Madrid, Spain; 8 Health and Nutrition Program, Federal University of Ouro Preto, Minas Gerais, Brazil; Universidade Federal de Santa Catarina, BRAZIL

## Abstract

The present study evaluated the factors associated with the perception of anxiety during the first wave of covid-19 in Ibero-American countries. This cross-sectional study was carried out with 5.845 participants of both sexes, over 18 years of age, and residents of four Latin American countries–Argentina (16.7%), Brazil (34.5%), Mexico (11.1%), and Peru (17.5%), and one European country–Spain (20.1%). Data were collected in 2020, between April 1st and June 30th in Spain and between July 13th and September 26th in the Latin American countries. We used an online questionnaire with sociodemographic, lifestyle, self-reported anxiety, and covid-19 related questions. The chi-square statistical test and Multivariate logistic regressions were performed to analyze the factors associated with self-reported anxiety. The presence of self-reported anxiety was found in 63.8% of the participants during the isolation period. The association occurred mainly in women (OR:1.52; CI: 1.3–1.7), those aged 18 to 29 years (OR: 1.51; CI: 1.2–1.9) and 30 to 49 years (OR: 1.56; CI: 1.3–1.9), residents of Argentina (OR: 1.55 CI: 1.2–1.9), Brazil (OR: 2.38; CI: 2.0–2.8) and Mexico (OR: 1.52; CI: 1.2–1.9), those who gained weight (OR:1.71 CI: 1.5–1.9) or lost weight (OR: 1.40; CI: 1.2–1.6), and those who reported having slept more (OR: 1.56; CI: 1.3–1.8) or less (OR: 2.89; CI: 2.5–3.4). We conclude that the prevalence of self-reported anxiety in Ibero-American countries was high during the period studied, highlighting a higher likelihood of its occurrence in Brazil, in those who began to sleep less and gained weight.

## Introduction

In March 2020, the World Health Organization declared the outbreak of Coronavirus 2019 (covid-19) a pandemic [1], which drove governments around the world to take different measures to avoid the spread of the virus, such as quarantines/confinement and social distancing [2]. Although these measures play a major role in preventing the collapse of the health system, they also affect the safety, well-being, and health of the individual and the community [3].

It is estimated that the pandemic has led to a 25.6% increase in the number of cases of anxiety disorder worldwide [4]. In 2017, around 264 million people worldwide were living with an anxiety disorder [5]. In measurements scale disability-adjusted life years (DALYs), the anxiety disorder, it ranked 34th in 1990, already 2019 it occupied the 24th position. [6]. Prior to the pandemic, problems related to mental health were already showing a growing and being the focus of concern, which led to the creation of “The Comprehensive Mental Health Action Plan 2013–2030” by the WHO in order the mental health is valued, promoted and protected [7].

Several studies have shown that confinement measures affected people in their home routines [8], sleep patterns [9, 10], screen time [8, 10], eating habits [9–11], and physical activity [10, 12]. Those measures also triggered concerns for the mental health of society as a whole [13]. Studies have shown that during the covid-19 pandemic a series of emotional reactions and unhealthy behaviors sprung in the general population, exposing some vulnerable groups to psychosocial effects, such as the older population and people with compromised immune function [3]. For instance, a high prevalence of negative mental health outcomes was reported in 1.210 respondents from 194 Chinese cities, with 16.5% reporting moderate to severe depressive symptoms and 28.8% reporting moderate to severe anxiety. In this study, 53.8% of the participants reported that the covid-19 outbreak caused a moderate or severe psychological impact [14]. Other studies showed that the number of adults with clinically significant symptoms of anxiety became 2 times higher than it was before the pandemic [15–17]. Socio-demographic aspects such as female gender, age, working conditions, and history of burnout or depression may influence the development of anxiety during the covid-19 pandemic [18].

A recent study conducted, in the USA, during the covid-19 pandemic revealed that US Americans consider mental illness as “severe” as other health-related conditions such as cancer, heart disease, and diabetes [19]. Therefore, identifying the factors involved in the development of mental health problems, such as anxiety, and their possible sequelae can direct mitigation strategies.

Fear of the unknown affects anxiety levels [20], during the pandemic the unknown was present, the symptoms were unknown, how serious the disease could be, the form of protection and whether there were effective remedies. Several studies have observed the association of anxiety during isolation with the pre-existence of some chronic diseases [21], intimate partner violence (IPV) [22], exposure to news about the disease [23], economic recessions, unemployment, decreased income and debt are directly associated with the decline in mental health [24].

Although some have reported the occurrence of mental health problems during the covid-19 pandemic in Latin American countries [25, 26]. There is a scarcity of comparative literature between Ibero-American countries, therefore, our study is one of the first to show evidence about this subject for comparison. The present study contributes to the planning and organization of action proposals for situations similar to that which occurred in the covid-19 pandemic. The health situation faced by these countries makes it possible to know the vulnerable contexts that can be of unique strategic value in addressing mental health issues. Thus, the present cross-sectional study seeks to describe the prevalence of self-perception of anxiety in adult individuals during the confinement imposed by the first covid-19 wave in Ibero-American countries, as well as to determine its associated factors.

## Methods

### Study design and population

This present study sample belongs to the project entitled “Lifestyle and eating habits in pandemic times: an Ibero-American study” [9], first applied in Spain denominated ALVIMED project (Analysis of the food consumption of families due to confinement following the covid-19 pandemic in the countries of the North and South of the Mediterranean basin and its impact on nutritional status), between April 01^st^ and June 30^th^, and right after it was done in the Latin American countries (Argentina, Brazil, Mexico and Peru), between July 13^th^ and September 26^th^. The survey collection methodology was the same in all participating countries.

The study project asked questions of perception about anxiety and life habits, retrospective and current (the first wave of confinement). The instrument was designed and culturally adapted by a team research experts. Briefly, these projects enrolled 6.325 individuals, adults over 18 years of age, of both sexes, through an online survey, which were under confinement during the first wave of covid-19.

Disclosure and invitation to participate in the study took place through social networks (e-mail, Facebook, Instagram, and WhatsApp) and were mediated by the participating universities in each country.

For the present analysis, we excluded 480 participants who did not have information about self-reported anxiety and confinement.

### Assessment of socio-demographic, health and well-being, and lifestyle exposures

The Google Forms web platform was used for data collection through a single structured self-administered questionnaire elaborated by a team of lifestyle research experts. The instrument was culturally and linguistically adapted for each studied country. The following socio-demographic variables were evaluated: gender (male and female), age group (18 to 29 years old, 30 to 49 years old, ≥ 50 years old), marital status (single, married, separated/divorced, widowed), schooling (high school education, college education, graduate), employment situation (student, worker, study and work, housewife, unemployed/retired), country of residence (Argentina, Brazil, Peru, Mexico, Spain). The questions related to lifestyle changes during the pandemic confinement included: *“Was there weight change during social distancing*?*”* (No change, I have kept my weight; Yes, I have gained weight; Yes, I have lost weight), *“Have you modified your sleep routine*, *during social distancing*?*”* (No change; Yes, I have slept more; Yes, I have slept less), *“During the period of confinement do you practice some type of physical activity*?*”* (Yes; No). The questions related to confinement and diagnosis of covid-19 included: “*Were you part of the confinement*?*”* (No; Yes, I am still confined; Yes, but I resumed my activities), *“Have you had a confirmed diagnosis of covid-19*?” (Yes; No). The question about self-perception of anxiety was: “*Has the social distancing situation caused you feelings of anxiety*?” (Yes; No).

### Ethical aspects

The instrument was applied to participants who agreed to take part in the study after clicking on the “accept” icon on the first page of the electronic invitation to participate in the study, meaning that they have agreed with the terms of the written informed consent. On this page, there was a brief description of the study and its goals.

This study was carried out in accordance with the principles of the Declaration of Helsinki and with the approval of the Ethics and Research Committees of each participating country: Research and Ethics Committee of the School of Medicine of the Del Plata Adventist University in Argentina (Resolution n° 1.7/2020); Ethics Committee of the Federal University of Espírito Santo in Brazil (approval n°: 33948820.7.0000.5060); Ethics Committee of the University of the Americas Puebla in Mexico (approval n° 019/2020); Research Ethics Committee of the Peruvian Union University (approval n° 2020-CEUPeU-00013); and Ethics Committee of the Autonomous University of Madrid, Spain (Project: Cohort UAM/AUF COVID-19 (CEI 106–2082).

### Statistical analysis

Categorical variables were analyzed using the chi-square statistical test to analyze the distribution and association of socio-demographic variables, lifestyle, confinement, diagnosis of covid-19, and self-reported anxiety. Binary logistic regression was performed to analyze the factors associated with the outcome (anxiety). The following models were evaluated: the raw model and adjusted model by sociodemographic variables (gender, age, education, marital status, and employment situation) and the lifestyle variables (weight, sleep, physical activity) and confinement variables. The results of the logistic regression analyses were expressed as odds ratios (OR) and 95% confidence intervals (95% CI). For all analyses, p ≤ 0.05 was considered significant. All statistical analyses were performed using the Stata 16.0 statistical software.

## Results

The study sample consisted of 5.845 participants, who answered the question whether confinement generated self-reported anxiety, of which 68.1% are female, almost half are between 18 and 29 years old (48.7%) and 56,9% are single. The study sample was mainly represented by those with a complete college education (42.4%), workers (36.9%), or both (15.1%). Of the five countries, the Brazilian sample was the largest (34.5%). A high proportion of participants reported a perception of weight gain (48.6%) and an increase in sleep (42.6%) during confinement. During the period of the data collection, 58.2% of the participants declared the practice of physical activity, 79.8% were confined and 93.5% did not have a positive diagnosis for covid-19 (Table 1).

**Table 1:** Sociodemographic characteristics, lifestyle, confinement, and diagnosis of covid-19 during the confinement due to the first wave of covid-19 and prevalence of anxiety in participants of the Ibero-American Study, 2020.

		Self-reported Anxiety		
Characteristics	Total	Yes	No	*p*
	No. (%)	No. (%)	No. (%)	
**Total**	5.845 (100%)	3.730(63.8)	2.115 (36.1)	
**Gender**				<0 .001
Female	4.018 (68.7)	2.696 (72.2)	1.322(62.5)	
Male	1.827 (31.2)	1.034 (27.7)	793(37.4)	
**Age group (years)**				< 0.001
18 to 29	2.850 (48.7)	1.885 (50.5)	965 (45.6)	
30 to 49	2.100 (35.9)	1.391 (37.2)	709 (35.5)	
≥ 50	895 (15.3)	454 (12.1)	441(20.8)	
**Marital status**				< 0.001
Single	3.325 (56.9)	2.177 (56.9)	1,148 (54.2)	
Married	2.182 (37.3)	1.347 (37.3)	835 (39.5)	
Separated/Divorced	271 (4.6)	170 (4.6)	101 (4.8)	
Widowed (o)	67 (1.1)	36 (0.9)	31 (1.4)	
**Schooling**				0.071
High school education	1.550 (26.5)	985 (24.4)	565 (26.7)	
College education	2.482 (42.4)	1.543 (41.3)	939 (44.4)	
Graduate	1.813 (31.0)	1.,202 (32.2)	611 (28.8)	
**Employment situation**				< 0.001
Student	2,143 (36.8)	1,448 (39,0)	695 (33.1)	
Worker	2,149 (36,9)	1.305 (35.1)	844 (40.1)	
Studies and works	879 (15.1)	601 (16,9)	278 (13.2)	
Housewife	160 (2.7)	107 (2,9)	53 (2.5)	
Unemployed/Retired	482 (8,3)	251 (6,7)	231 (10.9)	
**Country**				< 0.001
Argentina	978 (16.7)	687 (17.3)	361 (17.0)	
Brazil	2.016 (34.5)	1.467 (39.3)	549 (25.9)	
Peru	1.024 (17,5)	606 (17.5)	418 (19.7)	
Mexico	650 (11.1)	432 (11.6)	218 (10.3)	
Spain	1.177 (20.1)	608 (16.3)	569 (26.9)	
**Weight changes**				< 0.001
No, I have kept my weight	1.480 (27,7)	788 (23.1)	692 (35.8)	
Yes, I have gained weight	2.598 (48.6)	1.818 (53.3)	780 (40.3)	
Yes, I have lost weight	1.262 (23.6)	802 (23.5)	460 (23.8)	
Sleep changes*				< 0.001
No	1.568 (26.8)	783 (20.9)	785 (37.1)	
Yes, I have slept more	2.491 (42.6)	1.614 (43.2)	877 (41.4)	
Yes, I have slept less	1.785 (30.5)	1.333 (35.7)	452 (21.3)	
**Physical activity**				< 0.001
Yes	3.406 (58.2)	2.060 (55.2)	1.346 (63.6)	
No	2.439 (41.7)	1.670 (44.7)	8769 (36.3)	
**Confinement**				< 0.001
Yes, I am still confined	4.666 (79.8)	2.971 (79,6)	1.695 (80.1)	
Yes, but I resumed my activities	1.179 (20.1)	759 (20.3)	420 (19.8)	
**Covid-19 diagnosis**				<0.653
Yes	371 (6.3)	241 (6.4)	130 (6.1)	
No	5.468 (93.6)	3.485 (93.5)	1.983(93.9)	

*Different sample size

*p* Chi-square test

The prevalence of self-reported anxiety in this study was 63.8%, being predominant in females (72.2%), in individuals with a college degree (41.3%), single (56.9%), and in those who performed physical activity (55.2%). Most of them were confined (79.8%) and only 6.4% had a positive diagnosis for covid-19. The parameters gender, age group, marital status, employment situation, country, weight changes, sleep changes, physical activity, and confinement were significantly associated with the presence of self-reported anxiety (Table 1).

Table 2 shows the association between sociodemographic variables, lifestyle, and confinement with self-reported anxiety during the confinement due to the covid-19 pandemic through three multiple logistic regression models. We observed a positive association with self-reported anxiety in females, in those under 49 years of age, and in those who had a perception of weight and sleep changes. These last were more pronounced among those who reported weight gain and less sleep during confinement. Latin American individuals, particularly Brazilians, were more likely to self-reported anxiety when compared with Spaniards.

**Table 2:** Association of sociodemographic variables, lifestyle, and confinement among participants who have self-reported anxiety during the confinement due to the covid-19 pandemic in Argentina, Brazil. Peru, México and Spain 2020.

	Raw OR	Adjusted OR
Characteristics	(CI 95%)	(CI 95%)
**Gender**		
Female	**1.56 (1.4–1.7**)	**1.52 (1.3–1.7)**
Male	1	1
**Age group**		
18 to 29	**1.89 (1.6–2.2)**	**1.51 (1.2–1.9)**
30 to 49	**1.90 (1.6–2.2)**	**1.56 (1.3–1.9)**
≥ 50	1	1
**Marital status**		
Single	1	1
Married	0.85 (0.7–0.9)	1.06 (0.9–1.2)
Separated/Divorced	0.88 (0.7–1.4)	0.97 (0.7–1.3)
Widowed(a)	0.61 (0.4–1.0)	0.77 (0.4–1.3)
**Employment situation**		
Worker	1	1
Student	**1.34 (1.2–1.5)**	**1.21 (1.1–1.4**)
Studies and works	**1.39 (1.2–1.6)**	1.13 (0.9–1.3)
Housewife	1.30 (0.9–1.8)	1.29 (0.8–1.9)
Unemployed/Retired	0.70 (0.6–0.8)	0.95 (0.7–1.2)
**Country**		
Spain	1	1
Argentina	**1.59 (1.3–1.9)**	**1.55 (1.2–1.9)**
Brazil	**2.50 (2.1–2.9)**	**2.38 (2.0–2.8)**
Peru	**1.35 (1.1–1.6)**	**1.33 (1.2–1.9)**
Mexico	**1.85 (1.5–2.2)**	**1.52 (1.2–1.9)**
**Weight**		
No, I have kept my weight	1	1
Yes, I have gained weight	**2.04 (1.8–2.3)**	**1.71 (1.5–1.9)**
Yes, I have lost weight	**1.53 (1.3–1.8)**	**1.40 (1.2–1.6)**
**Sleep**		
No	1	1
Yes, I have slept more	**1.84 (1.6–2.0)**	**1.53 (1.3–1.8)**
Yes, I have slept less	**2.95 (2.5–3.4)**	**2.92 (2.5–3.4)**
**Physical activity**		
Yes	1	1
No	**1.41 (1.2–1.6)**	**1.21 (1.1–1.3)**
**Confinement**		
Yes, I am still confined	1	1
Yes, but I resumed my activities	**1.03 (1.1–1.1)**	**1.05 (1.1–1.2)**

Model 1: Raw mode + sociodemographic variables +

lifestyle variables (weight, sleep, physical activity) +

lifestyle variables (weight, sleep, physical activity) and confinement.

## Discussion

The findings of the present study showed that the self-perception of anxiety during the confinement due to the first wave of the covid-19 pandemic was more present in the female population, in the younger age group (≤ 49 years), and in individuals living in Latin American countries compared with those who lived in Spain. Moreover, self-reported anxiety was more prevalent among those who reported weight gain and less sleep time during the confinement compared with those who had not experienced weight or sleep changes, respectively.

An analysis performed with data from the United Kingdom Household Longitudinal Study (UKHLS) identified an increase in mental health-related problems during the pandemic, going from 23% in 2017–2019 to approximately 37% at the end of April 2020, particularly in women and young adults [27], corroborating our findings. According to Devoto et al. (2022), women are experiencing poor mental health as a result of the daily impact caused by the covid-19 pandemic [22]. That may be due to them assuming the role of primary caregivers within their families, which can make them more vulnerable to the increase in household activities following the closure of schools and other facilities [28].

A study in the United Kingdom showed that in homes with both parents, women suffered more interruption in paid work and loss of employment in order to take full responsibility for child care [29]. In addition, the outbreak of covid-19 and political restrictions have influenced the increased risk of IPV against women and created difficulties to report it [30]. Since the beginning of the pandemic, high rates of violence have been reported by women in Latin America and the Caribbean [31].

It is important to highlight that our sample was composed mostly of students, which is an important characteristic for the analysis of our outcome, since the change from face-to-face teaching to virtual teaching has described some disadvantages such as loss of contact, difficulty in maintaining academic integrity, lack of feedback, bored scenarios and lack of access to the Internet, aggravating social and educational inequalities [32]. In the same way in Italy it has been suggested that the success of online activities depends on ¨mental well-being¨ to face this new routine, showing that this context may contribute to high prevalence of anxiety [33].

Those under the age of 50 were more likely to feel anxiety, especially in the 30–49 age group. Our finding is consistent with other cross-sectional studies that have evaluated mental health in young adult populations during the covid-19 pandemic [34–36]. People in these age groups are generally employed and during the pandemic there was a major change in work style. One study suggests that the shift to remote work at home due to the covid-19 pandemic generated a greater influence on mental health, mainly due to increased workload and overtime [37]. Another study demonstrated relationships between drastic changes in work style, workplace, economic deterioration, feelings of job insecurity with anxiety symptoms [38]. In line with the WHO survey (2017), in which younger individuals had a higher prevalence of anxiety disorder, with a decline after 49 years old [5].

The relationship between physical activity and mental health has been studied by another research [39, 40]. Puccinelli et al., [40] identified a significant association between low levels of physical activity practice and higher incidences of anxiety and depression. A longitudinal study found that physical activity led to significantly improved symptoms of anxiety and depression [41]. The impact of the pandemic is clear and indisputable, the psychological consequences are significant. It has been found that periods of isolation, even shorter than 10 days, can affect mental health up to 3 years later, leading to the onset, in addition to anxiety, of insomnia, stress, depression, depressed mood, and others [42, 43].

According to pre-pandemic data, in 2017, 21% of the population that had an anxiety disorder was located in the Region of the Americas [5]. These pre-pandemic data of worsening anxiety in a study of adults that compared the presence of depression or anxiety in the first half of 2019 with that from April to May 2020 and found that anxiety was 3 times higher in this population during the pandemic than in 2019 [44]. A study estimated that the countries hit hardest by the pandemic in 2020, as measured with decreased human mobility and daily SAR-COV-2 infection rate, had the greatest increases in prevalence of major anxiety disorders [45]. In this same study, due to the covid-19 pandemic, the cases of anxiety disorders increased 76.2 million globally [45].

Among the countries analyzed, Brazil was the one with the highest odds of reporting anxiety during the covid-19 pandemic. According to Campos et al. (2020), in Brazil the prevalence rates of anxiety are as high as 44% at the end of 2020, compared to pre-pandemic frequencies [46] and another Brazilian study reported 81.9% prevalence of anxiety compared to other countries in Latin America [47]. According to Watkins-Martins et al. (2021), anxiety symptoms may vary according to socioeconomic policies, which differ between countries [48].

To Blackman et al. (2021), a coordinated, continuous, and coherent response to a crisis by a government is of paramount importance, especially as to full disclosure to citizens [49]. In Latin American countries, including Brazil and Peru, three types of measures were evaluated (mitigation and containment, health, and economic) to reduce the spread of covid-19. However, while Peru carried out mitigation and containment, and health measures rather quickly, Brazil took a while to implement measures such as confinement, local lockdown, school closures, and ban the gatherings [50]. The only measures implemented with less delay by Brazil were of economic nature. These measures came into force soon after the first confirmed case in each country, with Peru being the first to implement a national lockdown and Brazil the last to declare a state of emergency and shut its borders.

In Argentina, at the very beginning of the pandemic, the government implemented health actions and expanded the health emergency by ordering the adoption of measures to contain the spread of covid-19 through the Decree of Necessity and Urgency (DNU) No. 260/2020, established in March 2020. A study assessing psychological outcomes in the Argentinian population in April 2020, found that people assented to be confined even knowing that their income would be significantly affected [51]. These initial perceptions about the possible benefits of confinement for health reasons may have changed as strict restrictions were prolonged ensuing in psychological distress, symptoms of anxiety, and economic constraints in the Argentinian population.

In Spain, the government published the Decree-Law No. 21/2020 in June 2020, which described several urgent preventions, containment, and coordination measures necessary to face covid-19 to be applied in the country. Among such measures were prevention and hygiene, transport, and epidemiological surveillance measures, as well as the implementation of a lockdown all over Spain from March 28 until April 26, 2020 [52].

In Mexico, the scenario described by Ibarra-Nava et al. (2020) is similar to that observed in Brazil, where the impact of the covid-19 pandemic was minimized by the leader of the Mexican government [53]. According to Knaul et al. (2021), Brazil and Mexico were the epicenters of the pandemic in Latin America, lacking effective national leadership, essential functions such as disease surveillance, and public health statements to the population. In these countries, coherent guidelines were left to state and city governments, making combined intersectoral management unfeasible [54].

The lack of coordination regarding public policies in the face of the covid-19 pandemic may have been one of the reasons why Brazilians and Mexicans were at high risk of developing anxiety. This challenge could have been even greater in Brazil than in other countries due to its continental dimensions and diversity. Evidence-based public health measures such as mask use and social distancing were overlooked by the federal government. In addition, the population received conflicting and dubious messages [55], the government canceled the publication of epidemiological reports [50], and pandemic denial was present for a long time [8, 56].

In the present study, those with self-reported anxiety had a greater likelihood of suffering weight changes during the pandemic. It has been shown that individuals who experienced anxiety during the pandemic were significantly more likely to gain weight compared with those without anxiety (53% vs. 45%) [57]. Common mental disorders can be both a cause and a consequence of obesity and can be part of some mechanisms underlying this bidirectional relationship. If the covid-19 pandemic persists, adults will need to prevent weight gain and obesity by improving their mental health and lifestyle. More research is needed to elucidate the effects of weight gain during the covid-19 pandemic through large-scale data collection.

It has been already shown that outbreaks of infectious diseases are associated with psychological distress and poor sleep quality [58, 59]. The sleep changes found in this study, both sleeping more and sleeping less, were associated with self-reported anxiety, which demands attention, given the importance of sleep for health as a whole. The causal relationship between mental health and sleep has been discussed and asserted by several studies, as was the relationship between anxiety levels and poor sleep quality [60–63]. Sleep is essential because of its benefits for mental and physical health, as lack of sleep or poor sleep quality can cause psychological damage, compromise the immune response, increase the risk of accidents, and cause mood swings [64].

A meta-analysis found that the prevalence of sleep problems during the covid-19 pandemic is high, affecting around 40% of the general population [58]. In another study, the combined prevalence of anxiety was 47% (IC 95%: 37–57%, I2 = 97%) and that of sleep disorders was 34% (IC 95%: 19–50%, I2 = 98%). According to Blume et al. (2020) [65], significant changes in sleep were caused by the interruption of the subjects’ routine, and the reduction in the quality and quantity of sleep is consequently related to poor mental health outcomes [62, 63].

Some government actions aimed at meeting the population’s mental health demands should be carried out. Technology can be used as an instrument to access support services, teleconsultation, online support services, social media, etc. According to a systematic review and meta-analysis, the use of individual psychotherapies, peer support groups, breathing techniques, meditation and yoga contributed to the attenuation of anxiety disorders [66]. It is important that governments implement their legislation and fund programs dedicated to the mental health of their population [4], strengthening their systems and services of health, providing adequate resources for mental health and psychosocial support. The neglect of investments in this area, before the pandemic, made it difficult to respond appropriately to current needs [67].

The contributions of this study reinforce the observations made in previous studies [22, 34–37]. The present research may contribute to the planning and organization of action proposals for situations similar to what occurred in the covid-19 pandemic. Identifying sociodemographic and lifestyle characteristics related to self-reported anxiety, in order to mitigate the implications of confinement on mental health.

The results presented here must be regarded within the limitations of the present study, such as the cross-sectional design which leads to reverse causality, and convenience sampling which could call into question the external validity of the findings. The questionnaires were applied in a single moment and asked the participant to remember their lifestyle habits before the pandemic, which may present memory bias. Another limitation is that the participating countries were at different stages of the pandemic, which was also true for prevention measures and lockdowns. Therefore, the self-reported anxiety levels reflect the increase in anxiety as a result of the pandemic and may have been even higher in some places because of the public health measures implemented, or the lack of such measures. Tests for the diagnosis of covid-19, especially in the first year of the pandemic, were difficult to access and distribute, leading to a limitation in testing different populations [68, 69], which may have influenced the association investigated. In addition, as the instruments were self-reported, there may be variation in the assessment of symptoms in clinical interviews or by specialists, as self-reported tests do not necessarily translate a clinical symptom.

Nevertheless, considerable participation, based on the use of social networks, was achieved in each country. The questionnaires were not validated but were created at the beginning of the first wave of covid-19 and were tested by a panel of experts. Within the strengths of our study, the same questionnaires were applied in different countries of Ibero-american, with adequacy to language. In addition, design and implementation occurred during the social confinement phase of the pandemic in all countries. Nevertheless, we acknowledged some distinct factors among countries (e.g., data collection during spring and early summer in Spain, late summer in Mexico and late winter in South American countries).

Future studies should be carried out in order to identify the presence of self-reported anxiety in Ibero-American and other countries. For example, a cohort study used the Generalized Anxiety Disorder Scale-7 (GAD-7), and found that anxiety disorder increased comparing the time before and after the covid-19 pandemic [70]. Another study that used the Overall Anxiety Severity and Impairment Scale (OASIS) found anxiety owing to pandemic of covid-19 [71]. It could also serve as a better to guide possible prevention and optimize resources for the most vulnerable people within the overall population.

In conclusion, more than half of the analyzed population self-reported anxiety during the first wave of covid-19, with sociodemographic variables involving lifestyle, place of residence, weight gain, and less sleep as the main associated factors. In addition, we highlight the heterogeneous role played by political organizations at the beginning of the control measures. The actions taken by governments reverberated throughout the pandemic and made its consequences clear, being a negative example. In Latin America were implemented later, particularly in Brazil, which had the highest probability of presenting anxiety reports and the most flexible measures among the countries analyzed.

## Supporting information

S1 Database (ZIP)
