# Correction: Prevalence and factors associated with self-reported anxiety in adults during the COVID-19 pandemic in Argentina, Brazil, Peru, Mexico, and Spain: A cross-sectional Ibero-American study

**DOI:** 10.1371/journal.pone.0306214

**Published:** 2024-06-21

**Authors:** Gabriela Oliveira, Fernanda Garcia Gabira Miguez, Oscar G. Enríquez-Martinez, Taisa S. S. Pereira, Karen Villaseñor Lopez, Salomon Huancahuire-Vega, Marcia C. T. Martins, Sandaly O. S. Pacheco, Fabio J. Pacheco, María del Pilar Montero López, Maria del Carmen Bisi Molina

The tenth author’s name is written incorrectly. The correct name is: María del Pilar Montero López.
